# Brain MRI and regional vulnerabilities to radiation necrosis: investigating the impact of stereotactic radiotherapy in brain metastases treatment

**DOI:** 10.3389/fradi.2025.1554017

**Published:** 2025-04-09

**Authors:** Carlo A. Mallio, Ugo Ferrari, Gianfranco Di Gennaro, Matteo Pileri, Caterina Bernetti, Enrica Polo, Emma Gangemi, Francesca Giannetti, Paolo Matteucci, Bruno Beomonte Zobel, Edy Ippolito, Sara Ramella

**Affiliations:** ^1^ Fondazione Policlinico Universitario Campus Bio-Medico, Rome, Italy; ^2^ Research Unit of Radiology, Department of Medicine and Surgery, Università Campus Bio-Medico di Roma, Rome, Italy; ^3^ Department of Health Sciences, Chair of Medical Statistics, University of Catanzaro “Magna Græcia”, Catanzaro, Italy; ^4^ Department of Human Neurosciences, Sapienza University of Rome, Rome, Italy; ^5^ Research Unit of Radiation Oncology, Fondazione Policlinico Universitario Campus Bio-Medico, Rome, Italy

**Keywords:** brain MRI, radiation necrosis, stem cell niches, stereotactic radiotherapy, brain metastases

## Abstract

**Background:**

Radiation necrosis is a significant late adverse effect of stereotactic radiotherapy (fSRT) for brain metastases, characterized by inflammatory processes and necrotic degeneration of healthy brain tissue.

**Objective:**

To evaluate the relationship between the incidence of radiation necrosis and the distribution of lesions across different brain regions treated with fSRT, with a focus on the potential involvement of stem cell niches.

**Methods:**

We conducted a *post-hoc* analysis of two separate prospective datasets consisting of data from 41 patients previously treated for brain metastases at Campus Bio-Medico University Hospital. Patients underwent fSRT using volumetric-modulated arc radiotherapy (VMAT), with MRI data collected pre- and post-treatment. Lesions were assessed for the presence of radiation necrosis based on radiological and clinical criteria, with a specific focus on their proximity to stem cell niches. A mixed-effects logistic regression model, including age and sex as covariates, was used to identify associations between brain region, stem cell niches, and the likelihood of radiation necrosis.

**Results:**

Of 167 lesions observed, 42 (25.1%) were classified as radiation necrosis. The Deep-Periventricular region showed a significantly higher likelihood of radiation necrosis compared to other brain regions (log-OR: 1.25, 95% CI: 0.20–2.30, *p* = 0.02). Lesions in proximity to stem cell niches were significantly associated with an increased risk of radiation necrosis (log-OR: 1.61, 95% CI: 0.27–2.94, *p* = 0.018). These findings highlight the differential vulnerability of brain regions and suggest a potential role of stem cell niches in the pathogenesis of radiation necrosis.

**Conclusion:**

This study underscores the importance of brain region and stem cell niche involvement in the development of radiation necrosis following stereotactic radiotherapy. These findings might have implications for optimizing radiotherapy planning and developing targeted strategies to mitigate the risk of radiation necrosis. Future research should focus on exploring the molecular mechanisms underlying these associations and evaluating potential neuroprotective interventions.

## Introduction

Brain radiotherapy has an established role in the multidisciplinary treatment of brain metastases. Particularly radiosurgery (SRS) and fractionated stereotactic radiotherapy (fSRT), characterized by precise high-dose delivery that minimizes collateral damage to non-targeted tissues, have been increasingly used for the treatment of brain metastases, even if multiple (1–3). Radiation necrosis represents a late adverse effect of brain radiotherapy been mainly related to the injury of adjacent healthy brain tissue, resulting in inflammatory processes and necrotic degeneration of the parenchyma. The incidence of radiation necrosis is significant, with reported prevalence rates ranging from 5% to 50%, depending on the radiation dose and individual clinical factors (4, 5). Even though the pathophysiological mechanisms underlying radiation necrosis remain incompletely elucidated, literature suggests the involvement of vascular and glial damage (6). Experimental models have demonstrated that radiation can disrupt the permeability of the blood-brain barrier, leading to diffuse edema and reduced tissue oxygenation (7). This process triggers the activation of factors such as VEGF and HIF-1, which promote the proliferation of disorganized capillaries, further exacerbating necrosis (8).

Conventional MRI findings of radiation necrosis and tumor recurrence often overlap (9, 10). Therefore, advanced imaging modalities, such as diffusion tensor imaging, perfusion imaging, spectroscopy, and positron emission tomography, can aid in differentiating radiation necrosis from tumor recurrence (9, 10). Among these, perfusion imaging, particularly the assessment of curves and relative cerebral blood volume (rCBV) color-coded maps, is one of the most commonly applied MRI technique in clinical practice (9, 10).

Recent studies are exploring the potential involvement of brain stem cell niches in the neuroprotection and in the development of post-radiation neuroinflammation and cognitive disfunction (11–13). Specifically, the ventricular-subventricular zone (V-SVZ) and the sub-granular zone (SGZ) of the hippocampal dentate gyrus have been identified as critical regions due to their central role in neurogenesis and their susceptibility to radiation-induced damage (14). These niches are integral to cellular turnover and brain tissue regeneration, making them particularly vulnerable to injury, such as radiation-induced damage (11, 13, 14). Irradiation of areas adjacent to these neural territories has been shown to impair neurogenic functions, hinder tissue repair, and contribute to long-term memory deficits and cognitive dysfunction (12, 15, 16).

We hypothesize that the lesion location, also at the level of stem cell niches, may play a role in the development of radiation necrosis due to their involvement in neurogenesis and susceptibility to radiation-induced damage.

Thus, this study aims to evaluate the relationship between the incidence of radiation necrosis and the distribution, also in relation to stem cell niches, of lesions across different brain regions treated with stereotactic radiotherapy.

## Materials and methods

The study was designed as observational, and all the included patients were part of two prospective datasets enrolling patients affected by stage IV breast and lung cancer, approved by the Ethical Committee of the Fondazione Policlinico Universitario Campus Bio-Medico [N. PROT. PAR.: 004.23(16.19) and 73.23 OSS]. All the methods adhered to the Declaration of Helsinki's ethical principles, and all participants provided their signed informed permission.

### Inclusion and exclusion criteria

Patients treated and followed up at the Campus Bio-Medico University Hospital were included in the study. To be eligible for inclusion, patients had to meet the following criteria:

• Confirmed histological diagnosis of lung or breast primary tumor.
• At least one brain metastasis lesion identified through magnetic resonance imaging (MRI) of the brain, performed with and without contrast, treated with fSRT.
• Pretreatment and post-treatment MRIs both performed, at our hospital.

Exclusion criteria were:

• Patients who had undergone neurosurgical procedures.
• Patients with a history of previous brain irradiation, to minimize confounding effects.

### Radiation treatment

All patients included in the analysis underwent fSRT using volumetric-modulated arc-radiotherapy (VMAT) technique. Patients were immobilised with a thermoplastic mask and underwent 1 mm axial computed tomography (CT) scans for treatment planning calculation. A T1-weighted sequence (1 mm slice thickness) gadolinium enhanced MRI scan was fused to the simulation CT scan and used for gross tumor volume (GTV) target delineation. A margin of 3 mm was geometrically added to GTV to generate the planning target volume (PTV) to account for setup uncertainties. Total prescription doses were 27–30 Gy delivered in 3–5 fractions. Doses were prescribed to the 80% isodose line normalized to the maximum.

### MRI protocol

Brain MR images were obtained with a 1.5T MRI system (Magnetom Avanto B13; Siemens, Erlangen, Germany), and a 12-element head matrix coil. MRI protocol included axial FLAIR (repetition time [TR], 8.000 ms; echo time [TE], 102 ms; inversion time, 3.650 ms; matrix, 256 × 256; field of view [FOV], 26 × 30 cm; slice thickness, 3 mm), coronal TSE T2-weighted (TR, 6.380 ms; TE, 105 ms; matrix, 256 × 256; FOV, 26 cm × 30 cm; slice thickness, 3 mm), axial echo-planar DWI (TR, 3.927 ms; TE, 106 ms; matrix, 128 × 128; FOV, 23 cm × 23 cm; slice thickness, 5 mm; with diffusion sensitivity [*b*] = 0 and three orthogonal diffusion encoding gradients with *b* = 1,000), and axial T1-weighted spin echo images before and after intra-venous administration of gadolinium-based contrast agents (relaxation time [TR] 663 ms; echo time [TE] 11 msec; matrix, 256 × 256; field of view [FOV], 25 cm × 25 cm; slice thickness, 3 mm), together with a post-contrast fat suppressed T1 VIBE sequence (TR, 5.67 ms; TE, 2.38 ms; matrix, 512 × 256; FOV, 25 cm × 24.2 cm; slice thickness, 1 mm; voxel size, 0.5 × 0.5 × 1.0 mm³; 3D acquisition; fat suppression, yes) (17, 18). Dynamic susceptibility contrast (DSC) MR perfusion was also acquired.

### Imaging analysis

One neuroradiologist (CAM, 13 years of experience) and one resident in radiology (UF, 5 years of experience) evaluated the images by consensus and established the diagnosis and anatomic location of radiation necrosis. To this end, all the images were evaluated considering the available follow-up MRI studies together with clinical and therapeutic history, to establish whether radiological hallmarks of radiation necrosis were present or not (9, 19, 20). The MRI follow-up studies were performed with a mean time interval of 107 days.

**Figure 1:**
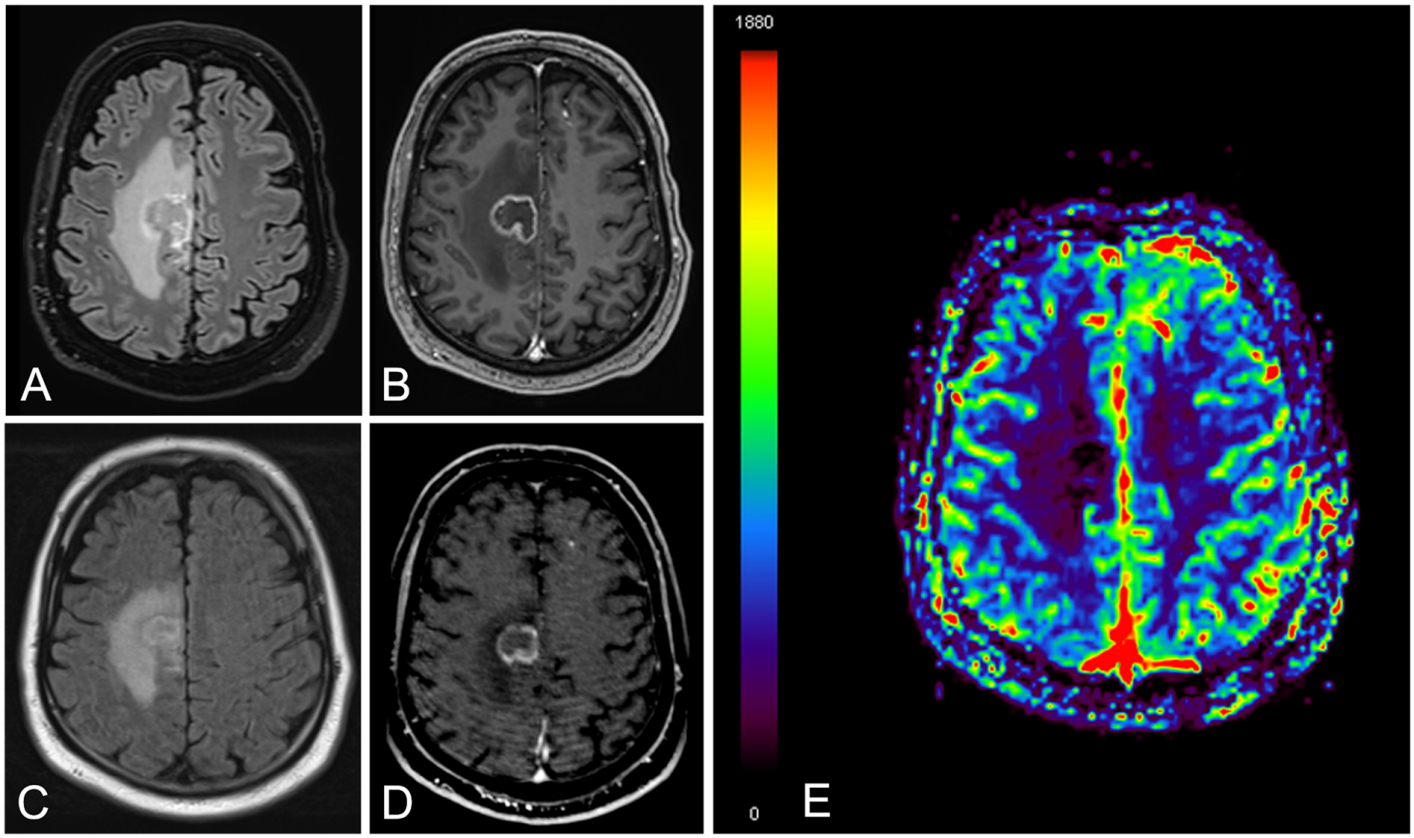
A 76-year-old male patient with brain metastasis and lung cancer. Brain MRI images obtained at eight months since stereotactic radiation therapy [**(A)** FLAIR and **(B)** contrast enhanced T1]. Follow-up MRI study at six months [**(C)** FLAIR, **(D)** contrast enhanced T1 and **(E)** relative cerebral blood volume map]. There is a right frontal ring enhancing lesion with peripheral vasogenic edema **(A–D)**, showing slight reduction in size at 6 months **(C,D)** together with lack of increased perfusion **(E)** The findings were consistent with radiation necrosis.

The lesion's evolution over time was closely monitored. Morphological changes were tracked through sequential imaging to detect signs of progression, stabilization, or regression. MRI criteria to establish the diagnosis of radiation necrosis were the following: T2/FLAIR high signal, peripheral vasogenic edema, early mass effect and subsequent loss of volume, absence of increased rCBV, and post-contrast ring-enhancement, “soap-bubble”, “cut green pepper” or “swiss-cheese” patterns (9, 10, 21, 22) (Figure 1).

These parameters provided a comprehensive framework for understanding the radiological presentation and behaviour of radiation necrosis over time.

Furthermore, the location of the lesions in proximity to the stem cell niches was also considered. The V-SVZ, located along the lateral wall of the lateral ventricles, and the SGZ nestled within the dentate gyrus of the hippocampus were meticulously evaluated (23).

### Statistical analysis

The data were described using mean and standard deviation for continuous variables with a normal distribution, and median with interquartile range for those with a skewed distribution. Categorical variables were summarized as counts and percentages. The normality of continuous variables was assessed using the Shapiro–Wilk test.

To identify potential differences in the likelihood of developing radiation necrosis across brain regions, a mixed-effects logistic regression analysis was performed, including age and sex as covariates. Patient ID was incorporated as a random intercept in the mixed-effects logistic regression model to account for within-patient correlation. Marginal probabilities of radiation necrosis were estimated for each brain region while keeping control variables constant.

An additional logistic model, also adjusted for sex and age, was used to investigate the relationship between stem cell niches and the presence of radionecrosis.

The analysis was conducted using STATA 16.0 software (https://www.stata.com), with a significance level set at 5%. This approach allowed for robust modeling of regional variability and the impact of key demographic factors on the development of radiation necrosis. Since the study is purely exploratory, multiple testing correction was not conducted.

## Results

A total of 41 patients were included in the analysis. In the sample of 41 patients, 24 were female (58.54%), while 17 were male (41.46%). The age of the patients followed a normal distribution (Shapiro–Wilk test; *p* = 0.789), with a mean of 61.83 years (SD: 9.41). Males were slightly older, with a mean age of 63.35 years (SD: 8.24), compared to females, who had a mean age of 61.74 years (SD: 9.17) (Table 1).

**Table 1:** Descriptive characteristics of the sample.

Demographics	Female (*n* = 24)	Male (*n* = 17)	Total (*n* = 41)
Percentage	58.54%	41.46%	100%
Mean Age (years)	61.74	63.35	61.83
Standard Deviation (years)	9.17	8.24	9.41

In the patient sample, the distribution of neoplasms was as follows: lung cancer accounted for 33 patients, representing about 80.5% of the total; breast cancer for 8 patients, representing about 19.5% of the total.

A total of 167 lesions were observed in 41 patients, localized in the four categories as described in Table 2. The median number of lesions per patient was 3, with an interquartile range of 1–6.

**Table 2:** Anatomical distribution of brain metastatic lesions.

Radionecrosis	Cortico-subcortical	Deep-periventricular	Cerebellar	Brainstem
No	77 (82.80%)	21 (60.00%)	24 (70.59%)	3 (60.00%)
Yes	16 (17.20%)	14 (40.00%)	10 (29.41%)	2 (40.00%)
Total	93 (100.00%)	35 (100.00%)	34 (100.00%)	5 (100.00%)

**Figure 2:**
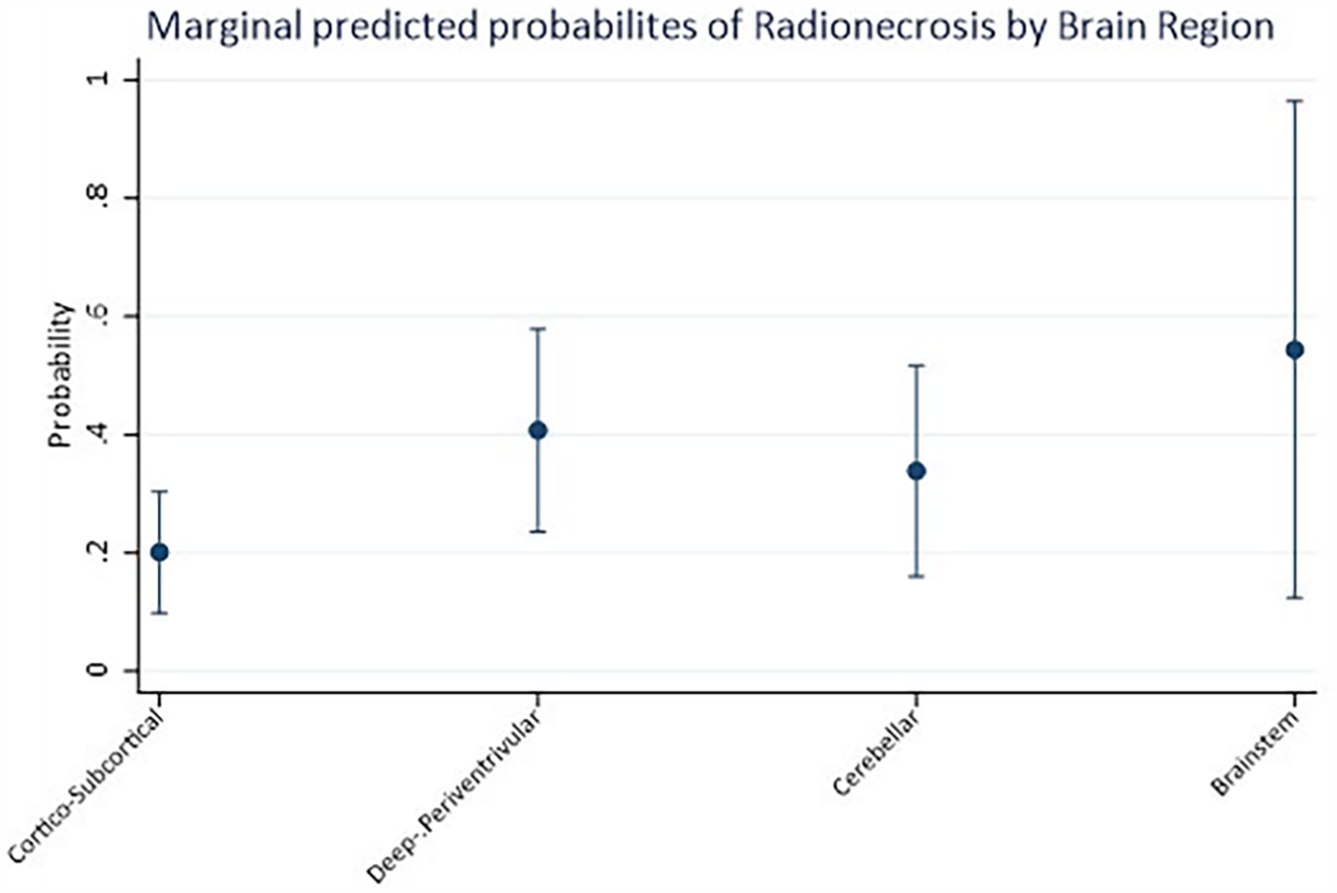
Marginal predicted probabilities of radionecrosis estimated by logistic regression analysis according to the anatomical distribution of brain metastatic lesions.

Mixed-effects logistic regression revealed a significant association between the brain region and the likelihood of radionecrosis. Indeed, compared to the cortico-subcortical region (reference category), the deep-periventricular location showed a log-odds ratio of 1.25 (95% CI: 0.20–2.30; *p* = 0.02), whereas the cerebellar and brainstem locations did not exhibit significant differences (Table 3). The marginal predicted probability of radionecrosis was 20.14% (95% CI: 9.85, 30.42%) in the cortico-subcortical region, 40.73 (95% CI: 23.54, 57.91%) in the deep-periventricular region, 33.87% (95% CI: 16.07, 51.68%) in the cerebellum and 54.40% (95% CI: 12.40, 96.40%) in the brainstem (Figure 2). Random-effects variance was = 1.37 (95% CI: 0.35, 5.28). Intraclass Correlation Coefficient was 0.29, indicating not negligible level of correlation within patients.

**Table 3:** Results of the logistic regression analysis according to the anatomical distribution of brain metastatic lesions.

Variable	Odds ratio	Std. err.	*z*	*P* > |*z*|	95% CI lower	95% CI upper
Age	0.99	0.03	−0.29	0.775	0.94	1.05
Gender	0.92	0.63	−0.13	0.897	0.27	3.16
Deep-periventricular	3.48	0.54	2.33	0.020	1.22	9.94
Cerebellar	2.40	0.57	1.53	0.125	0.78	7.36
Brainstem	7.03	1.16	1.68	0.093	7.21	68.40
Intercept	1.37	0.57	−0.64	0.523	0.01	11.57

Additionally, among the 42 suspected cases of radionecrosis, 6 cases (14.29%) were in regions compatible with stem cell niches. In contrast, among the 125 cases without radionecrosis, only 4 cases (3.2%) were observed in stem cell niche locations, resulting in a statistically significant difference (log-OR: 1.61; 95% CI: 0.27–2.94; *p* = 0.018) (Tables 4, 5).

**Table 4:** Anatomical distribution of brain metastatic lesions according to the stem cells niches.

Stem cells niches	No radionecrosis	Radionecrosis	Total
No	121 (96.8%)	36 (85.71%)	157 (94,00%)
Yes	4 (3.2%)	6 (14.29%)	10 (6.00%)
Total	125 (100.00%)	42 (100.00%)	167 (100.00%)

**Table 5:** Results of the logistic regression analysis according to the stem cells niches anatomical distribution of brain metastatic lesions.

Variable	Odds ratio	Std. err.	*z*	*P* > |*z*|	95% CI lower	95% CI upper
Stem cells niches	4.99	0.68	2.36	0.018	1.31	18.95
Age	1.02	0.03	0.49	0.625	0.95	1.09
Gender	1.48	0.69	0.56	0.572	0.38	5.69
Intercept	0.01	2.20	−2.06	0.040	0.0001	0.81

## Discussion

The study findings indicate a significant association between the deep-periventricular region and an increased risk of radionecrosis, highlighting a differential vulnerability of brain regions to radiation-induced damage. These results align with existing literature attributing a selective negative effect of radiation on specific brain areas, particularly those with higher vascular and neural density (4, 5). The susceptibility of the deep-periventricular region may be due to the complex interaction between vascular and neural tissue, leading to heightened reactivity to inflammatory stimuli induced by radiotherapy. Further analysis could clarify the role of local factors, such as microcirculation and blood-brain barrier regulation, in contributing to brain tissue impairment and post-irradiation necrotic degeneration (6).

Additionally, the relationship between radionecrosis and stem cell niches represents a particularly intriguing aspect. Stem cell niches, primarily located in the V-SVZ and the SGZ of the hippocampus, are critical areas for neural regeneration and brain plasticity. Recent studies suggest that these niches, through neurogenesis, may exert a protective influence against tissue damage, potentially mitigating the degenerative effects of radiation in certain brain regions (11–13). However, radiation exposure to these niches can disrupt their function, reducing regenerative capacity and inducing an inflammatory response that exacerbates tissue degeneration (11–13). These findings are consistent with previous research showing that vascular damage and impaired neurogenesis contribute to an increased risk of permanent damage following stereotactic irradiation (7).

The effect of radiation on stem cell niches underscores the importance of further clinical and experimental studies to examine the molecular and cellular mechanisms involved in greater detail. Chronic inflammation and microglial dysregulation are hypothesized to play a particularly significant role in the interaction between radiation-induced damage and stem cell niche function (8). These mechanisms might also influence patient responses to innovative treatments, such as VEGF pathway inhibitors or neuroprotective approaches aimed at preserving stem cell niche functionality during radiotherapy (1). Several preclinical studies suggest that selective inhibition of inflammatory or angiogenic pathways could reduce the incidence of radionecrosis, offering new perspectives for the treatment and prevention of radiation-induced damage.

Our findings align with prior studies, which also identified lesion location as a critical factor in radiation necrosis risk. Specifically, while we observed a higher risk associated with the deep-periventricular region and proximity to stem cell niches, previous research highlighted increased risk with larger metastasis volume, supratentorial locations, and specific histologies such as renal cell carcinoma (24). Additionally, our study complements prior findings by Choi et al. on radiation necrosis following stereotactic radiotherapy (25). They reported a significant association with deep white matter structures (zone 2, 36% incidence), followed by deep locations such as the brainstem and basal ganglia (zone 3, 16%). Both studies underscore the increased vulnerability of deep anatomical structures to radionecrosis, with our work focusing on the potential biological susceptibility related to neurogenic niches, and the study by Choi and colleagues categorizing risk by detailed anatomical zones, highlighting differences between cortical and deep regions (25). Together, these findings reinforce the need for precise radiotherapy planning to mitigate radionecrosis in high-risk areas.

This study has several limitations that should be acknowledged. The sample size was relatively small, which may limit the generability of the findings to larger populations. Furthermore, the data were collected from a single center, potentially introducing selection bias, and limiting the applicability of results to different settings or populations. Another limitation of this study is that only 10 lesions were located within stem cell niches, which, despite yielding a statistically significant association, necessitates cautious interpretation given the small number of events. Unmeasured confounding variables could have influenced the outcomes, and future prospective studies with larger, more diverse cohorts are needed to validate these findings and provide a more comprehensive understanding of the topic. Finally, a limitation of our study is the exclusion of tumor type as a covariate, which, despite the standardized treatment protocols and predominance of lung cancer in our cohort, may overlook potential histology-driven differences in radiation necrosis susceptibility.

Future research should focus on optimizing radiotherapy doses with respect to particularly sensitive brain regions, identifying radiological or molecular biomarkers for early risk assessment of radionecrosis in specific brain regions and exploring targeted interventions to modulate neurogenesis, aiming to mitigate the side effects of radiotherapy (3, 26).

## Conclusion

This study highlighted an association between specific brain regions and the likelihood of radionecrosis in the treatment of brain metastasis, as well as a potential correlation with stem cell niches. These findings could be valuable for the stratification of patients at higher risk of radionecrosis and the subsequent development of targeted preventive and therapeutic strategies in case of radiotherapy for brain metastasis.

## Data Availability

The raw data supporting the conclusions of this article will be made available by the authors, without undue reservation.
